# Critical attitudes and beliefs towards guidelines amongst palliative care professionals – results from a national survey

**DOI:** 10.1186/s12904-017-0187-y

**Published:** 2017-03-21

**Authors:** Helen Kalies, Rieke Schöttmer, Steffen T. Simon, Raymond Voltz, Alexander Crispin, Claudia Bausewein

**Affiliations:** 1 Department of Palliative Medicine, Munich University Hospital, LMU Munich, Marchioninistr. 15, 81377 Munich, Germany; 2 Center for Palliative Medicine, University of Cologne, Kerpener Str. 62, 50924 Köln, Germany; 3 Institute of Medical Informatics, Biometry and Epidemiology (IBE), University of Munich, Marchioninistr. 15, 81377 Munich, Germany

**Keywords:** Palliative care, Attitudes and beliefs, Guidelines, National survey, Germany

## Abstract

**Background:**

Little is known about palliative care professionals’ attitudes towards guidelines. In 2015, the German Association for Palliative Medicine (DGP) published an evidence based guideline for palliative care in adults with incurable cancer. Before publication we conducted a national survey among members of the DGP to detect possible barriers and facilitators for its implementation. The aim of the present publication was to evaluate critical attitudes and beliefs which could hinder the effective implementation of the new guideline and to evaluate differences within professional groups and medical specialisations.

**Methods:**

This web-based online survey was addressed to all members of the DGP in summer 2014. Twenty-one questions concerning attitudes and beliefs towards guidelines were a priori developed to represent the following topics: scepticism regarding the quality of guidelines, doubts about the implementation of guidelines, restrictions in treatment options through guidelines, discrepancy between palliative care values and guidelines. Differences within professions and specialisations were tested using Kruskal-Wallis tests.

**Results:**

All 4.786 members with known email address were invited, 1.181 followed the link, 1.138 began to answer the questionnaire and 1.031 completed the questionnaire. More than half of participating members were physicians and one third nurses. Scepticism regarding the quality of existing guidelines was high (range 12.8–73.2%). Doubts regarding practical aspects of guidelines were less prevalent but still high (range 21.8–57.6%). About one third (range 5.4–31.4%) think that guidelines restrict their treatment options. In addition, 38.8% believed that guidelines are a kind of cookbook and restrict the flexibility of individual patient care. The majority saw no or little discrepancy between palliative care values and guidelines (range 68.4–82.6%). There were relatively small but significant differences between professions and specialisations.

**Conclusion:**

The person-centred and individual approach of palliative care does not seem to contradict the acceptance of guidelines. Main barriers were related to scepticism regarding the quality of guidelines and the implementation of guidelines in general.

**Electronic supplementary material:**

The online version of this article (doi:10.1186/s12904-017-0187-y) contains supplementary material, which is available to authorized users.

## Background

Palliative care has gained increasing attention in recent years. It is defined as an approach which improves the quality of life of patients and their families facing life-threatening illness, through the prevention and relief of suffering by means of early identification and impeccable assessment and treatment of pain and other problems, physical, psychological and spiritual [1]. It is characterised by a holistic-philosophical approach, in which the individual needs of the patient play a major role [2]. Because of the patient’s particular vulnerability and the associated challenges concerning ethical aspects, scientific research is difficult to conduct. Therefore, evidence-based instructions are less common compared to other disciplines, such as internal medicine, oncology or cardiology, where in return the compliance rate with evidence based recommendations is higher than in other speciality areas [3]. As a result, scepticism towards and rejection of guidelines among palliative care professionals might be more likely than in other medical disciplines.

In May 2015, the German Association for Palliative Medicine (Deutsche Gesellschaft für Palliativmedizin; DGP) published in cooperation with the German Guideline Programme in Oncology and more than 50 institutions and experts an evidence and consensus based national guideline for palliative care in adults with incurable cancer [4]. The guideline focuses on key recommendations for breathlessness, pain, constipation, depression, communication, the dying phase and health structures across cancer diagnoses on the basis of the best scientific evidence. In contrast to many other guidelines, this palliative care guideline was designed for all professional groups working in oncology and palliative care and not only for physicians.

Although the development of guidelines is an important instrument to improve healthcare [5, 6], the sole publication of these guidelines will not lead to better health outcomes unless they are successfully implemented into routine practice [7–9]. The most effective implementation strategies are those that are specifically tailored to address previously identified barriers and enablers to change [10–12]. However, little is known about palliative care professionals’ attitudes towards guidelines. We therefore conducted a national survey among members of the German Association of Palliative Medicine before publication of the new guideline to detect possible barriers and facilitators for its implementation and to develop implementation strategies which could be deduced from these results. The participants were asked for their attitudes and beliefs concerning guidelines in general and in palliative care, their opinion concerning use and effect of existing recommendations in palliative care and assessment of their own competence in the key areas listed in the new guideline.

In the present paper we focus on attitudes and beliefs of members of the DGP towards guidelines in general and in palliative care. The aim was to

1. evaluate the prevalence of critical attitudes and beliefs which could hinder the effective implementation of the new guidelines in palliative care, and
2. evaluate differences in these critical attitudes within professional groups and medical specialisations.

## Methods

### Study population and design

This online survey was addressed to all members of the DGP. The DGP is a multi-professional and multidisciplinary association with about 5000 professionals representing all areas of palliative care.

The survey was conducted following the Checklist for Reporting Results of Internet E-Surveys (CHERRIES) [13]. It was programmed with SurveyMonkey (surveymonkey.com), a software tool for web-based surveys. This software allows various types of questions (open vs. closed; multiple vs. single answers; mandatory vs. optional answers). To ensure that every participant completed only one questionnaire, multiple use of the same IP address was denied; participants had the possibility to take a break and jump back.

In the run-up to this survey, members of the DGP were informed about the purpose of the survey in the regular online newsletter. Members with known email address were contacted and provided with more information about the study and a link to the survey. When clicking the link, they were connected to the SurveyMonkey starting page. This page provided information about the purpose of the study, the data protection rights and a possibility to terminate the participation and give reasons for non-participation. After one and two months, respectively, email reminders were sent. Data were collected from 2014-07-10 until 2014-09-15.

The questionnaire was based on a questionnaire developed to gain insight into general practitioners’ views on medical guidelines [14] and was adapted to guidelines in palliative care. The practicability and comprehensibility of the questionnaire was pre-tested in a pilot study with 27 palliative care professionals and adapted according to the feedback of the pilot participants. The final questionnaire included 62 questions on the following topics: attitudes towards guidelines in general (25) and in palliative care in particular (20); suggestions for the implementation of the future guideline in palliative care (1), beliefs about evidence-based medicine (6) and problems in the German healthcare system (8), and competence (1) and awareness (1) in five key areas of palliative care. There was also an option for free-text comments. Basic sociodemographic data (age, gender, profession, job specification, professional experience, type of workplace, estimated proportion of contact to seriously ill/dying patients) were also collected. Response categories ranged from a five-point ordinal scale to dichotomous and free text, respectively. The average processing time for the whole questionnaire was 15 min.

To increase participation, a price draw was offered to respondents with an iPad mini and books as winnings.

### Description of the variables

In this publication we focus on critical attitudes and beliefs concerning guidelines. Analyses of cognitive-behavioural barriers will be published separately. Out of all 45 questions concerning attitudes and beliefs towards guidelines in general and in palliative medicine in particular, 21 questions concerning critical attitudes were a priori chosen and grouped into the following barriers:

1. Scepticism regarding the quality of guidelines (7 questions)
2. Doubts about implementation of guidelines (6 questions)
3. Restriction in treatment options through guidelines (5 questions)
4. Discrepancy between palliative care values and guidelines (3 questions)

All 21 questions were originally categorised with a four-point Likert scale (do not agree, rather disagree, somewhat agree, agree; don’t know).

### Data analysis

The standard definitions of the American Association for Public Opinion Research, update 2015 [15], were used to define response, cooperation and refusal rates; we report the most conservative estimates (minimum rates), assuming that (1) email addresses are more or less kept up to date and non-respondents are therefore counted as those not willing to participate and partial (incomplete) questionnaires are not included in the nominator.

Data were described with their median, 25^th^ and 75^th^ percentiles or proportions. In Table 2, absolute and relative numbers of all answering members (*n* = 1031) are reported for the category “don’t know”, whereas relative frequencies were reported for the other 4 ordinal categories solely. For the latter, reference number was the remaining number of participants (*n* = 1031 minus number answering “don’t know”).

To describe the differences in possible barriers between professions and physicians’ specialisations, we summarised the questions of each of the four topics by (1) coding the four ordinal answer categories with a score between zero and three (0=“don’t agree”; 3=“agree”), summarising scores of all questions under one topic and, for better comparability between topics, standardising each of these resulting sums to reach a value between zero and three. Differences within palliative care professions and physicians’ specialisations were tested by using Kruskal-Wallis tests for independent samples based on p-values on the alpha level of 5%. If appropriate, pairwise comparisons were made.

All calculations were carried out with the software package SPSS Statistics 23. For the raw data, see Additional file 1.

## Results

### Response rates and description of the sample

Out of 4961 members of the DGP, 4786 had a known email address. Until 2014-09-15, a total of 1181 members followed the link to the survey, and 1138 began to answer it. The survey was completed by 1031 members. According to the above definitions [15], the response rate was 21.5% (1031/4786), cooperation rate 87.3% (1031/1181) and refusal rate 0.9% (43/4786), respectively.

The majority of participating DGP members was female and had a qualification in palliative care. More than half were physicians and one third nurses. The main other professions were: 19.7% social workers, 17.0% psychologists, 13.6% spiritual care workers and 10.2% physiotherapists. Among physicians, the main specialisations were: 26.4% anaesthesiology, 20.8% (general) internal medicine, 17.1% general medicine and 15.2% oncology. Almost half of all participating DGP members worked in a place specialised in palliative care (Table 1).

**Table 1:** Characteristics of the interviewed members of the German Association for Palliative Care DGP

	N	Median (25^th^–75^th^ Percentile)
Age (years)	1019	50 (44–54)
Professional experience (years)	1031	22 (15–29)
	N	Percent
Gender		
Female	665	64.5%
Male	366	35.5%
Profession		
Physician	572	55.5%
Among physicians:		
(general) internal medicine	119	20.8%
Oncology	87	15.2%
Anaesthesiology	151	26.4%
General medicine	98	17.1%
Other	117	13.0%
Nurses	312	30.3%
Other	147	14.3%
Undertaking research		
Yes	211	20.5%
No	820	79.5%
Qualification in palliative care/medicine		
Yes	885	85.5%
In process	70	6.8%
No	77	7.5%
Type of workplace		
Specialised palliative home care	164	15.9%
Hospice	54	5.2%
Hospital: palliative care unit	249	24.2%
Hospital: no palliative care unit	217	21.0%
Practices	143	13.9%
Ambulatory care service	30	2.9%
Science/research/teaching	67	6.5%
Other	107	10.4%

### Critical attitudes towards guidelines

A detailed description of the assessed critical attitudes among palliative care professionals is shown in Table 2.

**Table 2:** Prevalence of different critical attitudes towards guidelines (GL) amongst members of the German Association for Palliative Care DGP (*n* = 1031)

	Don’t know	Proportion of agreement (%)			
	N (%)	Disagree	Rather disagree	Somewhat agree	Agree
Scepticism regarding the quality of guidelines					
Credibility and independence of some authors of GL are questionable.	139 (13.5%)	13.2	45.1	26.7	15.0
GL are contradictory.	112 (10.9%)	27.6	53.0	13.8	5.5
GL are not always up to date.	50 (4.8%)	3.3	23.5	49.1	24.1
Legal position of guidelines is unclear.	165 (16.0%)	13.0	39.4	30.7	16.9
High number of GL is confusing.	47 (4.6%)	15.0	36.1	30.8	18.1
Too few studies to create GL in palliative care.	289 (28.0%)	13.6	40.2	36.3	10.0
Too little experience to create GL in palliative care.	92 (8.9%)	37.6	50.2	9.1	3.2
Doubts about implementation of guidelines					
GL are developed by experts who know little about everyday practice.	74 (7.2%)	28.8	49.3	16.3	5.5
It is difficult to change routine treatments.	18 (1.7%)	14.6	27.8	39.8	17.8
There are general problems in the application of GL.	80 (7.8%)	12.8	46.4	28.7	12.1
There are no incentives to apply GL.	57 (5.5%)	29.2	46.9	18.2	5.7
The use of GL is complicated.	40 (3.9%)	23.1	55.5	16.5	4.8
GL are too general and not concrete enough for day-to-day routines.	47 (4.6%)	19.3	55.4	18.6	6.7
Guidelines restrict treatment options					
GL are a kind of “cookbook medicine”.	38 (3.7%)	25.6	35.6	26.7	12.1
GL restrict the flexibility of individual patient care.	5 (0.5%)	26.5	42.1	18.9	12.5
GL restrict the physician’s therapeutic freedom.	34 (3.3%)	34.2	44.0	12.9	8.8
GL restrict one’s own thinking.	10 (1.0%)	41.7	42.2	9.4	6.7
GL question my own competence as physician/nurse.	17 (1.6%)	61.5	33.1	3.2	2.2
Discrepancy between values in palliative care and guidelines					
Evidence based medicine in GL contradicts the holistic philosophy of palliative care.	57 (5.5%)	37.4	46.0	12.7	3.9
GL are used for quality control. Quality control and dying must not be combined with each other.	38 (3.7%)	29.7	38.7	16.1	15.5
Palliative care GL restrict the individual healthcare of patients.	46 (4.5%)	32.1	50.6	12.1	5.3

Overall, scepticism regarding the quality of existing guidelines was high, with 73.2% of participants (somewhat) agreeing that guidelines are not always up to date; and more than 40% each (somewhat) agreeing that the independence of some authors is questionable, they are uncertain about the legal position, the amount of guidelines is confusing and that there are too few studies to create guidelines in palliative care. Interestingly, the proportion of participating DGP members stating “don’t know” was relatively high in this topic (up to 28% for “too few studies to create guidelines in palliative care”).

Doubts regarding practical aspects of guidelines were less common: 57.6% of participants (rather) agreed that it is difficult to change routine treatments, 40.8% that there are general problems in the application of guidelines and only 21.3% that the use of guidelines is complicated.

In contrary to the above results, more than two third of participating DGP members did not think that guidelines restrict their treatment options: 31.4% (somewhat) agreed that guidelines restrict the flexibility of individual patient care, 16.1% that they restrict their own thinking and only 5.4% that guidelines question their own competence. Nevertheless, more than one third (somewhat) agreed that guidelines are a kind of cookbook and restrict the flexibility of individual patient care.

In addition, the majority of participating DGP members saw no or little discrepancy between palliative care values and guidelines. Approx. 85% of participants did not think that evidence based medicine contradicts the holistic philosophy of palliative care or that palliative care guidelines restrict the individual healthcare of patients. The highest prevalence of critical attitudes in this topic was seen for the statement questioning the combination of quality control and dying (31.6% (somewhat) agreed).

### Differences by profession and specialisations

**Fig. 1:**
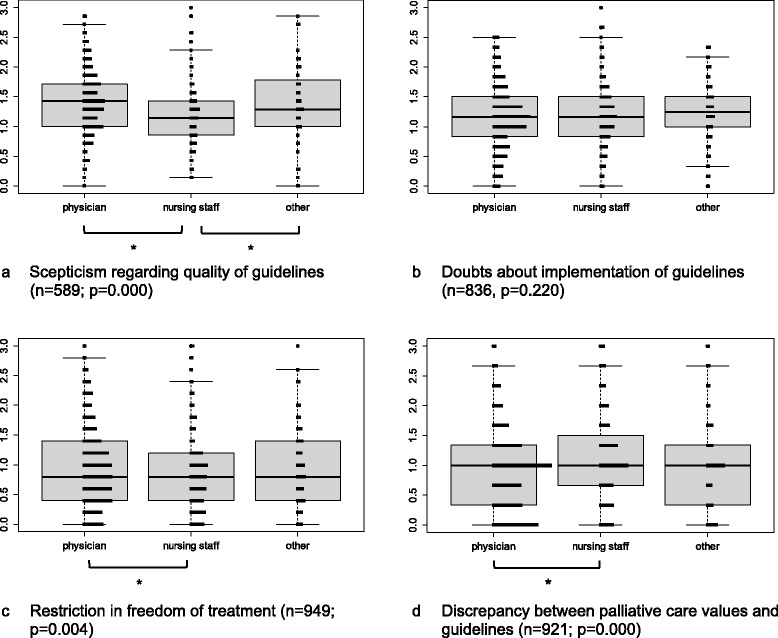
**a**–**d** Critical attitudes towards guidelines by profession and type of barrier amongst DGP members. Y-axis represents values from 0 (do not agree) to 3 (agree) for the summarised questions by topic. Boxplots (median, 25 and 75% percentiles; whiskers: 1.5 interquartile range) are shown together with a frequency distribution. *Asterisks* show significant (*p* < 0.05) pairwise comparisons for those outcomes with significant overall tests (*p* < 0.05)

There were relatively small but significant differences between professions in three of the four critical attitudes’ topics (Fig. 1a-d): Nurses were less sceptical regarding the quality of guidelines compared to physicians (*p* = 0.000) and other professions (*p* = 0.031) and had fewer concerns that guidelines restrict the freedom of individual treatment compared to physicians (*p* = 0.002). In contrast, nurses felt a slightly higher degree of discrepancy between palliative care values and guidelines compared to physicians (*p* = 0.000).

**Fig. 2:**
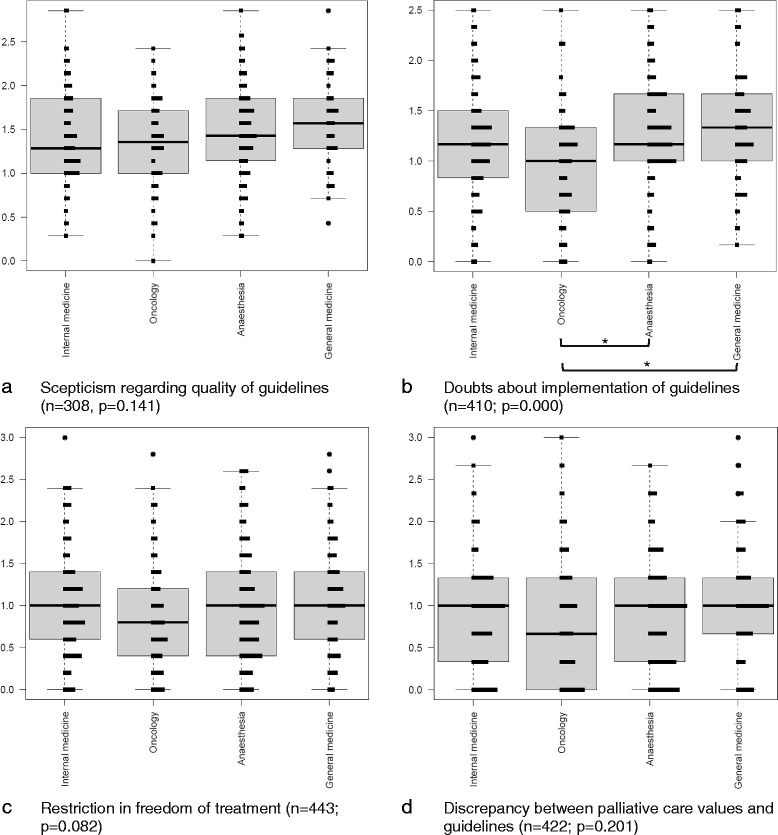
**a**–**d** Critical attitudes towards guidelines by physicians’ specialisation and type of barrier amongst DGP members. Y-axis represents values from 0 (do not agree) to 3 (agree) for the summarised questions by topic. Boxplots (median, 25 and 75% percentiles; whiskers: 1.5 interquartile range) are shown together with a frequency distribution. *Asterisks* show significant (*p* < 0.05) pairwise comparisons for those outcomes with significant overall tests (*p* < 0.05)

Among physicians, the only topic with critical attitudes showing significant (*p* = 0.000) differences among specialisations was “doubts about implementation of guidelines” (Fig. 2a-d): oncologists had less doubts than anaesthesiologists (*p* = 0.001) and general practitioners (*p* = 0.000), respectively.

## Discussion

This survey aimed to evaluate critical attitudes and beliefs of German palliative care professionals towards guidelines. We found that the individual and person-centred approach in palliative care does not automatically contradict the use of guidelines. Identified barriers and critical attitudes relate more to the quality and the implementation of guidelines in general. We believe that it is easier to overcome these barriers rather than a general negative attitude that the palliative care concept is not in accordance with the use of guidelines.

### Prevalence of critical attitudes and beliefs

Our study on palliative care professionals shows an overall similar dominance of positive attitudes and beliefs concerning guidelines compared to results found in other studies outside palliative care [14, 16–29], which focus on physicians [14, 16–18, 23, 25–32], nurses [21, 22] or both [19, 20, 24]. We did not find any data published for palliative care professionals’ attitudes towards guidelines in general.

The high scepticism about the quality of guidelines in general and the high proportion of undecided professionals in this topic underscores the need to make the process of the development of the new German guideline on palliative care for adults with incurable cancer transparent. More than 40% of participants in our survey agreed that the independence of some authors is questionable; this fits in the picture found by Butzlaff et al. [27] who report in a national German survey among ambulatory care physicians that the acceptance of guidelines from governmental institutions was substantially lower than from physician networks or medical societies. Earlier studies associated guideline adherence with participation in guideline construction [33]. Since the new guideline was developed by an interdisciplinary team from over 50 different institutions, with special emphasis on its evidence- and consensus-based character, a good balance of 120 practical and theoretical experts and the goal of regular updates, we think that these scepticisms can be reduced.

A high proportion of palliative care professionals had reservations about the transfer of guidelines into practice. Forty-one percent had general problems in the application of guidelines, the main specific doubt was the difficulty to change already existing routine treatments (57%). Other doubts regarding practical aspects of guideline implementation were not assessed, but lack of time and medical resources were reported by others [23, 25, 29]. In an online survey from Estonia, Taba et al. [29] showed that time was the barrier identified by most physicians (42%), followed by lack of medical resources for implementation (32%).

Although there was relatively little fear that guidelines restrict the palliative care professionals’ own thinking and questions their competence there were major concerns about their autonomy: More than one third of palliative care professionals reported fear that guidelines are a kind of “cookbook” and one fifth that they restrict their therapeutic freedom. In a review by Farquar et al. [19], similar results were found for “cookbook medicine” (34%) and higher for physician’s autonomy (34%). Most studies from the US, Canada and Australia [32, 34–37] show less, whereas studies from Germany show even higher concerns [14, 16] compared to our study, with an exception by Butzlaff et al. [27] (28% for “cookbook medicine”). An essential issue when implementing guidelines is therefore clarification what guidelines are: they are recommendations for action and “systematically developed statements which reflect the present state of knowledge to assist health professionals and patients in decision-finding for an adequate care in specific illnesses” [38]. They act as a guidance which can be altered in justified circumstances [39].

Because of its holistic-philosophical approach and the so far limited use of evidence-based recommendations a perceived discrepancy between values in palliative care and guidelines would have been one of the most difficult barriers to respond to. Nevertheless, the majority of palliative care professionals had little concern that guidelines in palliative care contradict the philosophy of palliative care.

Another aspect of acceptance of a guideline in palliative care could be general stigmata associated with palliative care in professionals outside classical palliative care institutions: A survey of the National Comprehensive Cancer Network in the US showed that “attitudes towards palliative care” are one of the major barriers for implementing clinical practice guidelines for palliative care in their member institutions [40]. In addition, Hui et al. [41] showed that an important clinician-related barrier is the stigma associated with the service name “palliative care”.

### Stratification by profession and medical specialisation

Differences between health care professions and various medical specialties towards guidelines in general have been described by others, with nurses [20, 24] having more positive attitudes than physicians and significant differences between general physicians and other medical specialists, but the direction of these differences was inconsistent [18, 29, 42].

In a national telephone survey with 511 participants, Butzlaff et al. [27] reported that German general practitioners agreed significantly more often with the usefulness of guidelines as a basis for patient care than specialists. In our study, we neither found relevant differences between physicians, nurses and other professions nor between physicians’ specialisations, with the exception of oncologists: they have a more positive attitude towards guidelines probably due to the fact that they are more used to guidelines [43]. Whether there are relevant differences in the use of guidelines and knowledge about topics addressed in the new guideline on palliative care is subject of further studies.

### Strengths and limitations

Although there were a number of quantitative surveys published since 2000 on health professionals’ attitudes towards guidelines in Asia [23], Australia [44], the US and Canada [20, 24, 32, 34, 45], and Europe [18, 21, 22, 25, 29, 46], there were only four nationwide studies on health professionals’ attitudes towards guidelines published in Germany [16, 26, 27, 47]. To our knowledge, this was the first study investigating palliative care professionals’ attitudes. In addition, we investigated attitudes of different professions and specialities so that comparisons between professional groups were possible and – in case of relevant differences – could be answered with restricted and tailored implementation strategies.

The relatively low response rate of 21.5% as calculated by the formula of the American Association for Public Opinion Research [15] can be judged as conservative, since it considers all non-respondents as if they had received and read the invitation email which is certainly only partly correct. A less conservative approach would lead to a slightly higher, but still low response rate of approx. 30% [48]. Nevertheless, the response rate is comparable to other online surveys among health professionals [49, 50] and to other German surveys [14, 16, 27, 51, 52]. A low response rate does not automatically translate into a low validity of survey results unless non-response bias was adequately considered [50, 53]. A comparison of all registered 4961 member of the DGP regarding routinely existing data, such as age, gender and profession, showed that the survey participants did not differ significantly in age and gender distribution and proportion of participating profession (data not shown here). The high cooperation rate and the low refusal rate also demonstrate that addressed members usually had no problem to complete the whole questionnaire as soon as they have clicked the link.

Generalisation of our results on all professionals working in the field of palliative care is difficult and there are two uncertainties: first, professionals in palliative care who are not members of the DGP and therefore were difficult to reach might have less positive attitudes towards guidelines than those organized in the DGP. Second, there is a variety of other medical specialisations working in generalist palliative care without having a specific qualification; this group might have more positive attitudes towards guidelines since they are used to the existence of guidelines through their originate professional society.

## Conclusion

The person-centred and individual approach of palliative care does not seem to contradict the acceptance of guidelines. Palliative care professionals’ attitudes towards guidelines are similar to other medical disciplines. Main barriers were related to scepticism regarding the quality of guidelines and doubts about the implementation of guidelines in general. High quality of the palliative care guideline will be achieved through evidence and consensus based recommendations and a thorough development process as recommended by the Guidance for Guideline Development of the Association of the Scientific Medical Societies in Germany. The implementation of the palliative care guideline needs to be a multi-stage approach with publications, presentations, courses, use of quality indicators and discussions with relevant stake holders.

## Additional file

Additional file 1: Data set supporting the results. (XLSX 100 kb)

## Abbreviations

AWMF: “Arbeitsgemeinschaft der Wissenschaftlichen Medizinischen Fachgesellschaften e.V.” - Association of the Scientific Medical Societies
DGP: “Deutsche Gesellschaft für Palliativmedizin” - German Association for Palliative Care

## References

[1] World Health Organization (2002). Definition of palliative care.

[2] Brueckner T, Schumacher M, Schneider N (2009). Palliative care for older people - exploring the views of doctors and nurses from different fields in Germany. BMC Palliat Care.

[3] Grilli R, Lomas J (1994). Evaluating the message: the relationship between compliance rate and the subject of a practice guideline. Med Care.

[4] S3- Leitlinie Palliativmedizin für Patienten mit einer nicht heilbaren Krebserkrankung. http://leitlinienprogramm-onkologie.de/Palliativmedizin.80.0.html. Accessed 2 Mar 2017.

[5] Woolf SH, Grol R, Hutchinson A, Eccles M, Grimshaw J (1999). Clinical guidelines: potential benefits, limitations, and harms of clinical guidelines. BMJ (Clinical research ed).

[6] Grimshaw JM, Russell IT (1993). Effect of clinical guidelines on medical practice: a systematic review of rigorous evaluations. Lancet.

[7] Lobach DF, Hammond WE (1997). Computerized decision support based on a clinical practice guideline improves compliance with care standards. Am J Med.

[8] Haines A, Donald A (1998). Making better use of research findings. BMJ (Clinical research ed).

[9] Craig P, Dieppe P, Macintyre S, Michie S, Nazareth I, Petticrew M (2008). Developing and evaluating complex interventions: the new Medical Research Council guidance. BMJ.

[10] Grimshaw JM, Eccles MP, Lavis JN, Hill SJ, Squires JE (2012). Knowledge translation of research findings. Implement Sci.

[11] Baker R, Camosso-Stefinovic J, Gillies C, Shaw EJ, Cheater F, Flottorp S, Robertson N (2010). Tailored interventions to overcome identified barriers to change: effects on professional practice and health care outcomes. Cochrane Database Syst Rev (Online).

[12] Prior M, Guerin M, Grimmer-Somers K (2008). The effectiveness of clinical guideline implementation strategies--a synthesis of systematic review findings. J Eval Clin Pract.

[13] Eysenbach G (2004). Improving the quality of Web surveys: the checklist for reporting results of internet E-surveys (CHERRIES). J Med Internet Res.

[14] Kunz AU. Leitlinien in der Medizin: Anwendung, Einstellungen und Barrieren - Eine Befragung Berliner Hausärzte. Masters Thesis, Freie Universität Berlin; 2005. http://www.ewi-psy.fuberlin.de/einrichtungen/arbeitsbereiche/ppg/media/projekte/hausaerzte/kunz_2005.pdf. Accessed 2 Mar 2017.

[15] Research TAAfPO (2015). Standard definitions: final dispositions of case codes and outcome rates for surveys.

[16] Larisch A, Oertel WH, Eggert K (2009). Attitudes and barriers to clinical practice guidelines in general and to the guideline on Parkinson's disease. A National Survey of German neurologists in private practice. J Neurol.

[17] Watkins C, Harvey I, Langley C, Gray S, Faulkner A (1999). General practitioners’ use of guidelines in the consultation and their attitudes to them. Br J Gen Pract.

[18] Carlsen B, Bringedal B (2011). Attitudes to clinical guidelines--do GPs differ from other medical doctors?. BMJ Qual Saf.

[19] Farquhar CM, Kofa EW, Slutsky JR (2002). Clinicians’ attitudes to clinical practice guidelines: a systematic review. Med J Aust.

[20] Sinuff T, Eva KW, Meade M, Dodek P, Heyland D, Cook D (2007). Clinical practice guidelines in the intensive care unit: a survey of Canadian clinicians’ attitudes. Can J Anaesth.

[21] Alanen S, Kaila M, Valimaki M (2009). Attitudes toward guidelines in Finnish primary care nursing: a questionnaire survey. Worldviews Evid Based Nurs.

[22] Kuronen R, Jallinoja P, Patja K (2011). Use of and attitudes toward current care guidelines among primary and secondary care nurses in Finland. Clin Nurs Res.

[23] Kwon H-M, Oh MS, Choi H-Y, Cho AH, Hong K-S, Yu K-H, Bae H-J, Lee J, Lee B-C (2014). Physicians’ attitudes toward guidelines for stroke: a survey of Korean neurologists. J Stroke.

[24] Quiros D, Lin S, Larson EL (2007). Attitudes toward practice guidelines among intensive care unit personnel: a cross-sectional anonymous survey. Heart Lung.

[25] Heselmans A, Donceel P, Aertgeerts B, Van de Velde S, Ramaekers D (2009). The attitude of Belgian social insurance physicians towards evidence-based practice and clinical practice guidelines. BMC Fam Pract.

[26] Hasenbein U, Schulze A, Busse R, Wallesch CW (2005). Physicians’ attitudes concerning guidelines. An empirical survey in neurologic clinics. Gesundheitswesen.

[27] Butzlaff M, Kempkens D, Schnee M, Dieterle WE, Bocken J, Rieger MA (2006). German ambulatory care physicians’ perspectives on clinical guidelines - a national survey. BMC Fam Pract.

[28] Hayward RS, Guyatt GH, Moore KA, McKibbon KA, Carter AO (1997). Canadian physicians’ attitudes about and preferences regarding clinical practice guidelines. Can Med Assoc J.

[29] Taba P, Rosenthal M, Habicht J, Tarien H, Mathiesen M, Hill S, Bero L (2012). Barriers and facilitators to the implementation of clinical practice guidelines: a cross-sectional survey among physicians in Estonia. BMC Health Serv Res.

[30] Grilli R, Penna A, Zola P, Liberati A (1996). Physicians’ view of practice guidelines. A survey of Italian physicians. Soc Sci Med.

[31] Grilli R, Trisolini R, Labianca R, Zola P (1999). Evolution of physicians’ attitudes towards practice guidelines. J Health Serv Res Policy.

[32] Graham ID, Evans WK, Logan D, O’Connor A, Palda V, McAuley L, Brouwers M, Harrison MB (2000). Canadian oncologists and clinical practice guidelines: a national survey of attitudes and reported use. Provincial Lung Disease Site Group of Cancer Care Ontario. Oncology.

[33] Grimshaw JM, Russell IT (1994). Achieving health gain through clinical guidelines II: Ensuring guidelines change medical practice. Qual Health Care.

[34] Wolfe RM, Sharp LK, Wang RM (2004). Family physicians’ opinions and attitudes to three clinical practice guidelines. J Am Board Fam Pract.

[35] Ferrier BM, Woodward CA, Cohen M, Williams AP (1996). Clinical practice guidelines. New-to-practice family physicians’ attitudes. Can Fam Physician.

[36] Gupta L, Ward JE, Hayward RS (1997). Clinical practice guidelines in general practice: a national survey of recall, attitudes and impact. Med J Aust.

[37] Tunis SR, Hayward RS, Wilson MC, Rubin HR, Bass EB, Johnston M, Steinberg EP (1994). Internists’ attitudes about clinical practice guidelines. Ann Intern Med.

[38] Field MJ, Lohr KN, Guidelines IoMCtAtPHSoCP (1990). Clinical practice guidelines: directions for a new program.

[39] German Association of the Scientific Medical Societies (AWMF) -Standing Guidelines Commission. AWMF Guidance Manual and Rules for Guideline Development, 1st Edition 2012. English version. Available at: http://www.awmf.org/leitlinien/awmf-regelwerk.html. Accessed 2 Mar 2017.

[40] Albizu-Rivera A, Portman DG, Thirlwell S, Codada SN, Donovan KA. Implementation of NCCN Palliative Care Guidelines by member institutions. Support Care Cancer. 2016;21(1):110–8. doi:10.1634/theoncologist.2015-0234. 10.1007/s00520-015-2862-y 26227917

[41] Hui D, Park M, Liu D, Reddy A, Dalal S, Bruera E (2015). Attitudes and beliefs toward supportive and palliative care referral among hematologic and solid tumor oncology specialists. Oncologist.

[42] Christakis DA, Rivara FP (1998). Pediatricians’ awareness of and attitudes about four clinical practice guidelines. Pediatrics.

[43] Langer T, Follmann M (2015). The German Guideline Program in Oncology (GGPO): A central core of an evidence-based, patient-centered interdisciplinary oncology?. Z Evid Fortbild Qual Gesundhwes.

[44] Lovell M, Agar M, Luckett T, Davidson PM, Green A, Clayton J (2013). Australian survey of current practice and guideline use in adult cancer pain assessment and management: perspectives of palliative care physicians. J Palliat Med.

[45] Grove A, Clarke A, Currie G (2015). The barriers and facilitators to the implementation of clinical guidance in elective orthopaedic surgery: a qualitative study protocol. Implement Sci.

[46] Formoso G, Liberati A, Magrini N (2001). Practice guidelines: Useful and “participative” method?: survey of italian physicians by professional setting. Arch Intern Med.

[47] Karbach U, Schubert I, Hagemeister J, Ernstmann N, Pfaff H, Hopp HW (2011). Physicians’ knowledge of and compliance with guidelines: an exploratory study in cardiovascular diseases. Dtsch Arztebl Int.

[48] Dobrow MJ, Orchard MC, Golden B, Holowaty E, Paszat L, Brown AD, Sullivan T (2008). Response audit of an internet survey of health care providers and administrators: implications for determination of response rates. J Med Internet Res.

[49] Hanbury A, Farley K, Thompson C (2015). Cost and feasibility: an exploratory case study comparing use of a literature review method with questionnaires, interviews and focus groups to identify barriers for a behaviour–change intervention. BMC Health Serv Res.

[50] Cho YI, Johnson TP, VanGeest JB (2013). Enhancing surveys of health care professionals: a meta-analysis of techniques to improve response. Eval Health Prof.

[51] Hagemeister J, Schneider CA, Barabas S, Schadt R, Wassmer G, Mager G, Pfaff H, Hopp HW (2001). Hypertension guidelines and their limitations--the impact of physicians’ compliance as evaluated by guideline awareness. J Hypertens.

[52] Meining A, Driesnack U, Classen M, Rosch T (2002). Management of gastroesophageal reflux disease in primary care: results of a survey in 2 areas in Germany. Z Gastroenterol.

[53] Johnson TP, Wislar JS (2012). Response rates and nonresponse errors in surveys. JAMA.

